# The effect of magneto-crystalline anisotropy on the properties of hard and soft magnetic ferrite nanoparticles

**DOI:** 10.3762/bjnano.10.133

**Published:** 2019-07-03

**Authors:** Hajar Jalili, Bagher Aslibeiki, Ali Ghotbi Varzaneh, Volodymyr A Chernenko

**Affiliations:** 1 Department of Physics, University of Tabriz, Tabriz 51666-16471, Iran; 2 Department of Physics, Isfahan University of Technology, Isfahan, 84156-83111, Iran; 3 BCMaterials and University of Basque Country (UPV/EHU), Sarriena s/n, Leioa 48940, Spain; 4 Ikerbasque, Basque Foundation for Science, 48013, Bilbao, Spain

**Keywords:** anisotropy, cobalt, ferrite, Henkel plots, hyperthermia therapy, nanoparticles, Rietveld refinement

## Abstract

Recent advances in the field of magnetic materials emphasize that the development of new and useful magnetic nanoparticles (NPs) requires an accurate and fundamental understanding of their collective magnetic behavior. Studies show that the magnetic properties are strongly affected by the magnetic anisotropy of NPs and by interparticle interactions that are the result of the collective magnetic behavior of NPs. Here we study these effects in more detail. For this purpose, we prepared Co*_x_*Fe_3−_*_x_*O_4_ NPs, with *x* = 0–1 in steps of 0.2, from soft magnetic (Fe_3_O_4_) to hard magnetic (CoFe_2_O_4_) ferrite, with a significant variation of the magnetic anisotropy. The phase purity and the formation of crystalline NPs with a spinel structure were confirmed through Rietveld refinement. The effect of Co doping on structure, morphology and magnetic properties of Co*_x_*Fe_3−_*_x_*O_4_ samples was investigated. In particular, we examined the interparticle interactions in the samples by δ*m* graphs and Henkel plots that have not been reported before in literature. Finally, we studied the hyperthermia properties and observed that the heat efficiency of soft Fe_3_O_4_ is about 4 times larger than that of hard CoFe_2_O_4_ ferrite, which was attributed to the high coercive field of samples compared with the external field amplitude.

## Introduction

Technological advances in various fields have motivated the design and the fabrication of nanostructures with tuned and improved properties. Among nanostructured materials, magnetic nanoparticles (NPs) are interesting from both fundamental and technological points of view [1–2]. In recent years, ferrite nanoparticles with the general formula of MFe_2_O_4_ (M = Fe, Co, Ni, Mn) have attracted great attention of researchers due to their potential applications in biomedicine and industry [3]. Magnetic anisotropy and interparticle interactions are important parameters that affect the magnetic properties and application fields of ferrite nanoparticles [1,4]. For example, NPs to be applied for data storage or magnetic recording must have a high coercivity, which is directly related to their magnetic anisotropy (the high coercivity keeps the recorded bits from being demagnetized) [5–6]. Magnetic interactions (e.g., exchange and dipolar interactions) have a strong effect on the magnetic behavior of a NP system (e.g., coercivity and blocking temperature) [7–8] and its potential for different applications. For example, there may be unfavorable effects in biomedical applications, such as aggregation of nanoparticles in different parts of the body [9]. Hence, the study of this kind of interactions is of particular importance, both from a practical and a fundamental point of view. Recently, Muscas et al. [1] studied the magnetic behavior of mixed cobalt–nickel and pure cobalt ferrite NPs by using a random anisotropy model. Their results showed that the overall magnetic properties are the equilibrium of the interplay between the interparticle interactions and the anisotropy of the single particles. The authors of this paper believe that this study is of fundamental importance to understand the physics of nanoparticle ensembles, which, in turn, is needed to develop technological applications of these systems. Among ferrites, CoFe_2_O_4_ NPs are of considerable interest because of their moderate saturation magnetization, good chemical stability and high intrinsic magnetocrystalline anisotropy at room temperature [10]. The anisotropy constant of CoFe_2_O_4_ (*K* = 2 × 10^5^ J·m^−3^) is nearly one order of magnitude larger than that of Fe_3_O_4_ [11–13]. Fe_3_O_4_ NPs have been studied extensively for bio-medical applications, such as drug delivery [14], magnetic resonance imaging (MRI) and especially magnetic hyperthermia therapy, which is one of the efficient and new approaches for cancer treatment [4,15]. When magnetic NPs concentrated in tumor tissue are exposed to an ac magnetic field, the electromagnetic energy is converted into thermal energy, and the generated heat is used to destroy cancer cells through the elevated temperatures [16–17]. The heating efficiency of the NPs as heat sources under ac magnetic fields is often denominated as specific absorption rate (SAR), which is directly related to the area of the magnetic hysteresis loop of the nanoparticles by the following formula [18–19]:

[1]
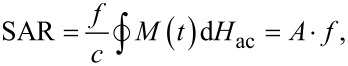


where *f* is the field frequency, *c* is the weight concentration of the material and *A* is the area of the hysteresis loop. Size and shape of the particles, saturation magnetization and magnetic anisotropy, as well as field amplitude and frequency strongly affect the hyperthermia output of a NP system [15,20–21]. Sathya et al. prepared Co*_x_*Fe_3−_*_x_*O_4_ nanocubes by a thermal decomposition method and showed that nanoparticles of 18–20 nm in size and a Co fraction of *x* = 0.5–0.7 have the highest SAR value and are suitable for hyperthermia applications [12]. Nemati et al. prepared iron oxide nanodiscs and compared their heating efficiency with spherical NPs of similar volume at different field strengths [21]. Their results indicated that the heating efficiency obtained for spherical nanoparticles is smaller than that measured for nanodiscs of similar volumes, especially at low field strengths. Barrera et al. prepared Co_1−_*_x_*Zn*_x_*Fe_2_O_4_ NPs and studied the dynamic energy losses of nanoparticles under an extended range of applied magnetic field strengths. They show that NPs with a larger anisotropy reveal smaller energy losses [22]. For a more systematic study of the effect of magnetic anisotropy and magnetic interactions on properties of magnetic nanoparticles, in this work, a series of Co*_x_*Fe_3−_*_x_*O_4_ (0 ≤ *x* ≤ 1) NPs was synthesized using a co-precipitation method. The effect of Co doping on the structural, magnetic and hyperthermia properties of Co*_x_*Fe_3−_*_x_*O_4_ nanoparticles has been studied. We report a detailed study of the magnetic interactions in the samples through field-dependent measurements of remanent magnetization. In order to investigate the magnetic interactions the Henkel plot method was used, which is an effective and powerful method.

## Results and Discussion

### X-ray diffraction

**Figure 1:**
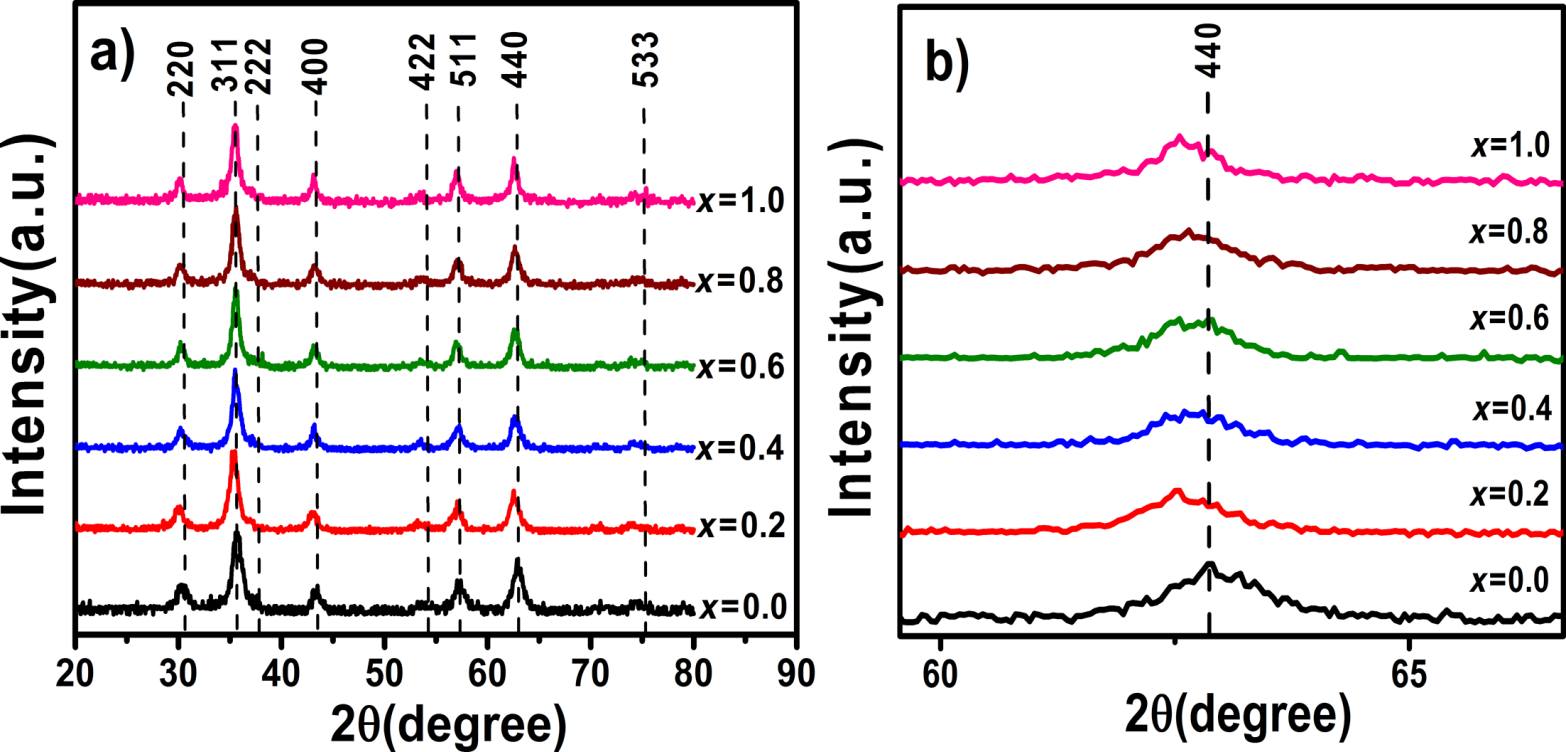
(a) XRD patterns of the Co*_x_*Fe_3−_*_x_*O_4_ (0 ≤ *x* ≤1) nanoparticles. (b) Shift of the (440) reflection.

The phase purity of the samples was confirmed by X-ray diffraction (XRD) analysis. Figure 1a shows XRD patterns of Co*_x_*Fe_3−_*_x_*O_4_ (0 ≤ *x* ≤1) nanoparticles. No secondary phases are found. The peaks intensities indicate that the samples are highly crystalline. The peaks match well with JCPDS cards (no. 01-088-0866 for Fe ferrite; no. 01-088-2152 for Fe–Co ferrites and no. 01-079-1744 for Co ferrite) indicating the formation of a cubic spinel structure with the space group *Fd*−3*m* (no. 227). Figure 1b showas that the representative (440) reflection shifts towards lower angles with cobalt ions increasingly substituting iron ions in the magnetite structure. This indicates an increase of the interplane distances (*d*) in the spinel structure. Similar results have been reported for Ni*_x_*Co_1−_*_x_*Fe_2_O_4_ NPs by Caetano and co-workers [15].

According to Bragg’s law, λ = 2*d*·sin θ, (λ is the wavelength of X-ray wavelength, here λ = 0.154 nm, and θ is the diffraction angle), a shift of θ to lower values indicates an increasing lattice spacing *d* that is directly related to the lattice constant *a* as:

[2] $$\begin{array}{c} a=d\sqrt{\left(h^{2}+k^{2}+l^{2}\right)}\;,\\ V_{\text{uc}}=a^{3}\;, \end{array}$$

where *h*, *k*, *l* are the Miller indices. The values of *a* and and the unit-cell volume *V*_uc_ for all samples were calculated by Equation 2 and are listed in Table 1. These values are in good agreement with the values reported before for Co*_x_*Fe_3−_*_x_*O_4_ (0 ≤ *x* ≤1) nanoparticles [23].

**Table 1:** Lattice constant (*a*), volume of unit cell (*V*_uc_), crystallite size ⟨*D*⟩_XRD_ and mean particle size ⟨*D*⟩_SEM_.

parameter	*x* = 0.0	*x* = 0.2	*x* = 0.4	*x* = 0.6	*x* = 0.8	*x* = 1.0

*a* (Å) (from Equation 2)	8.36(4)	8.40(1)	8.37(1)	8.38(3)	8.37(2)	8.40(1)
*a* (Å) (from Rietveld refinement)	8.346	8.406	8.379	8.382	8.373	8.399
*V*_uc_ (Å^3^) (from Equation 2)	584(8)	593(2)	586(2)	588(6)	586(4)	593(2)
*V*_uc_ (Å^3^)( Rietveld )	581.3	594.0	588.3	588.9	587.0	592.5
⟨*D*⟩_XRD_ (nm)	7.5 ± 0.8	8.6 ± 1.0	9.5 ± 2.0	10.3 ± 0.8	9.7 ± 1.1	13.1 ± 1.7
⟨*D*⟩_SEM_ (nm)	40.3 ± 8.5	28.7 ± 7.4	31.5 ± 6.1	25.8 ± 6.2	24.0 ± 5.0	27.1 ± 6.5

The radii of Co^2+^ ions (A-site: 58 Å, B-site: 74 Å) are slightly different than those of either Fe^2+^ ions (A-site: 61 Å, B-site: 78 Å) or Fe^3+^ ions (A-site: 49 Å, B-site: 64 Å) [24]. Therefore, the unsystematic variations and insignificant (in the error range) difference in the lattice constant could be attributed to the change of the cation distribution in the A-and B-sites.

**Figure 2:**
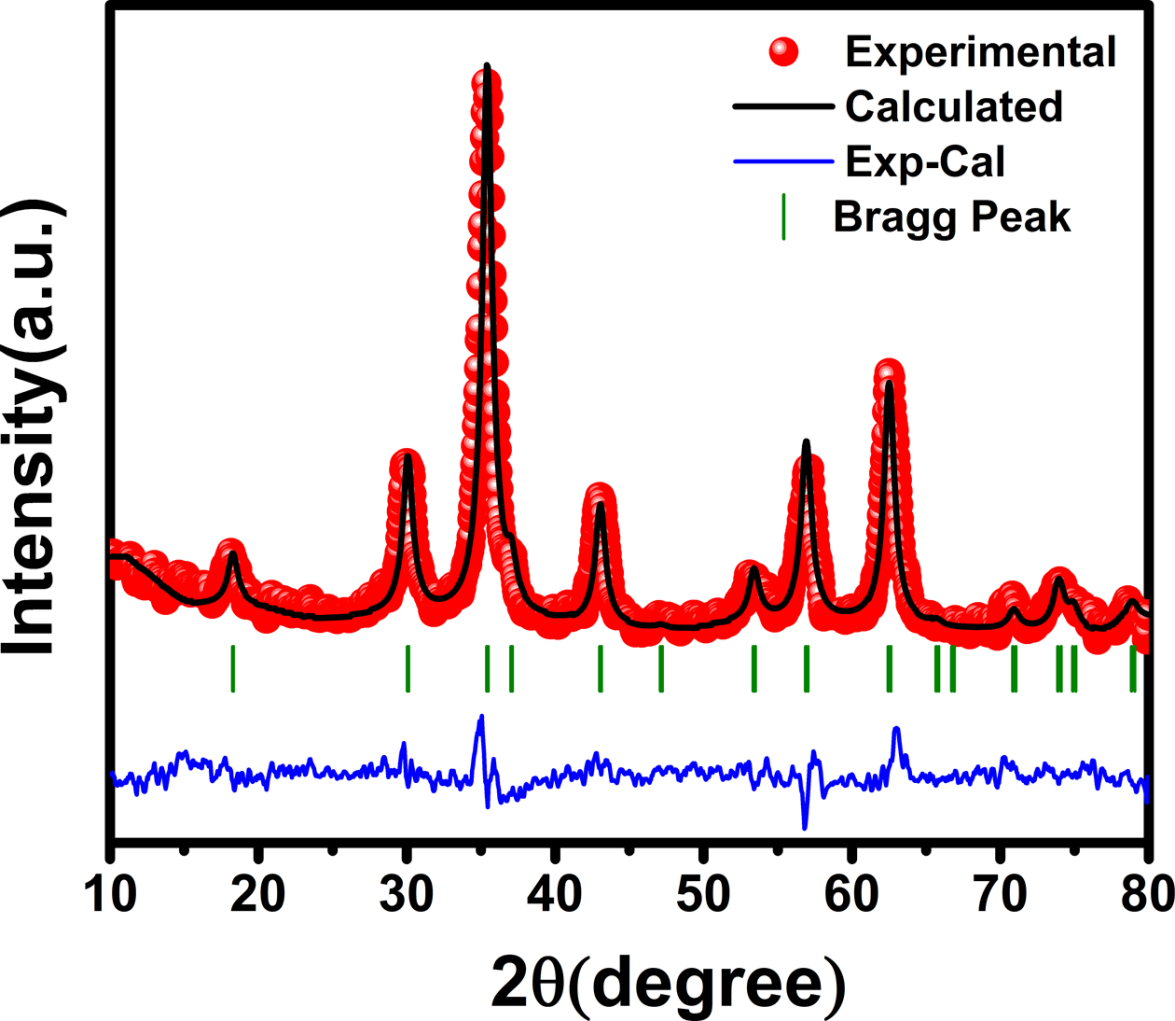
Rietveld-refined XRD pattern of the *x* = 0.2 sample.

The XRD patterns of the samples were analyzed using the Rietveld refinement method implemented in the the “FullProf Suite” software. As an example, the Rietveld refinement pattern of the Co_0.2_Fe_2.8_O_4_ sample is shown in Figure 2. Table 1 shows that the parameters *a* and *V*_uc_ obtained from Rietveld refinement are in good agreement with those calculated with Equation 2.

The effect of Co doping on the average crystallite size was studied using the Scherrer equation:

[3] $${\langle D\rangle }_{\text{XRD}}=\frac{K\lambda }{\beta \cos\theta },$$

where ⟨*D*⟩_XRD_ is the average crystallite size, *K* ≈ 0.9 is the Scherrer constant and β is the full width at half-maximum (FWHM) of the XRD peaks. Table 1 shows that the crystallite size increases with increasing cobalt content. The increase of the crystallite size is attributed to the bond energy of Co–O (397 kJ/mol), which is smaller than that of Fe–O (407 kJ/mol) [25]. The smaller bond energy speeds up the crystallization process, thus increasing the crystallite size in the samples.

### Microstructure and morphology

**Figure 3:**
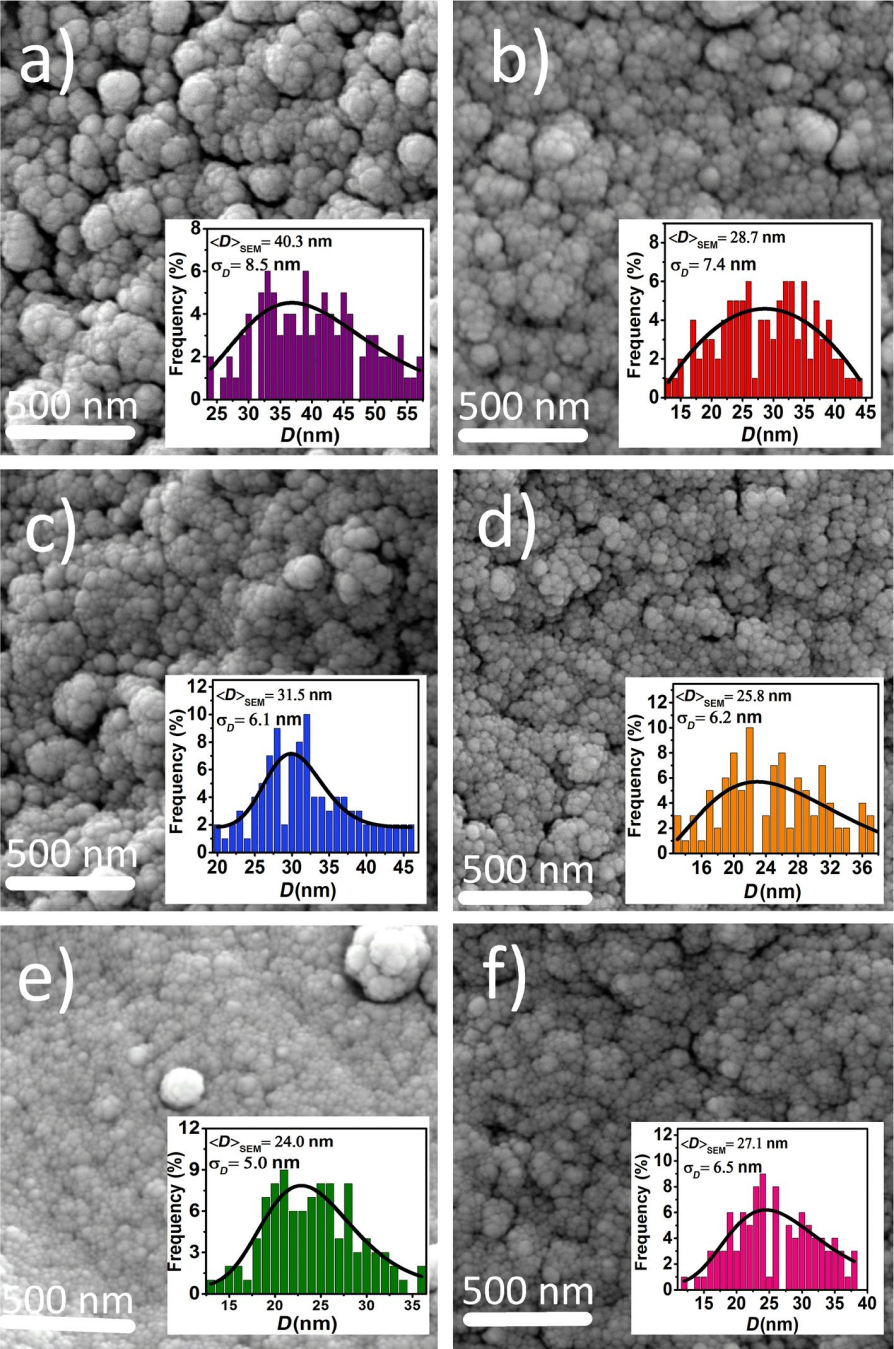
FE-SEM images of Co*_x_*Fe_3−_*_x_*O_4_ nanoparticles: (a) *x* = 0.0; (b) *x* = 0.2; (c) *x* = 0.4; (d) *x* = 0.6; (e) *x* = 0.8 and (f) *x* = 1.0. Insets show the particle size distribution fitted with a log-normal function (solid line).

In order to determine the particle size distribution and morphology of the samples, field-emission scanning electron microscopy (FE-SEM) was carried out. Figure 3 shows FE-SEM images of all the samples. The images reveal that particles are in the nanometer range and roughly spherical in shape. The particles size distribution in the samples was determined by measuring the size of 100 particles from the FE-SEM images fitting the size histogram with a log-normal function:

[4] $$f\left(D\right)=\frac{1}{\sqrt{2\pi }\sigma D}\exp\left[-\frac{\ln^{2}\left(D/D_{0}\right)}{2\sigma^{2}}\right],$$

where *D*_0_ is the median diameter and σ is the dispersion. The mean diameter ⟨*D*⟩ = *D*_0_·exp(σ^2^/2) and standard deviation σ_D_ = ⟨*D*⟩·[exp(σ^2^) – 1]^1/2^ were determined using the fit parameters *D*_0_ and σ and are given in Table 1. The obtained values of ⟨*D*⟩_SEM_ are larger than those obtained from the XRD patterns, which could be attributed to aggregation of the nanoparticles due to the presence of magnetic interactions between nanoparticles. The magnetic interactions are discussed in more detail in the following sections.

**Figure 4:**
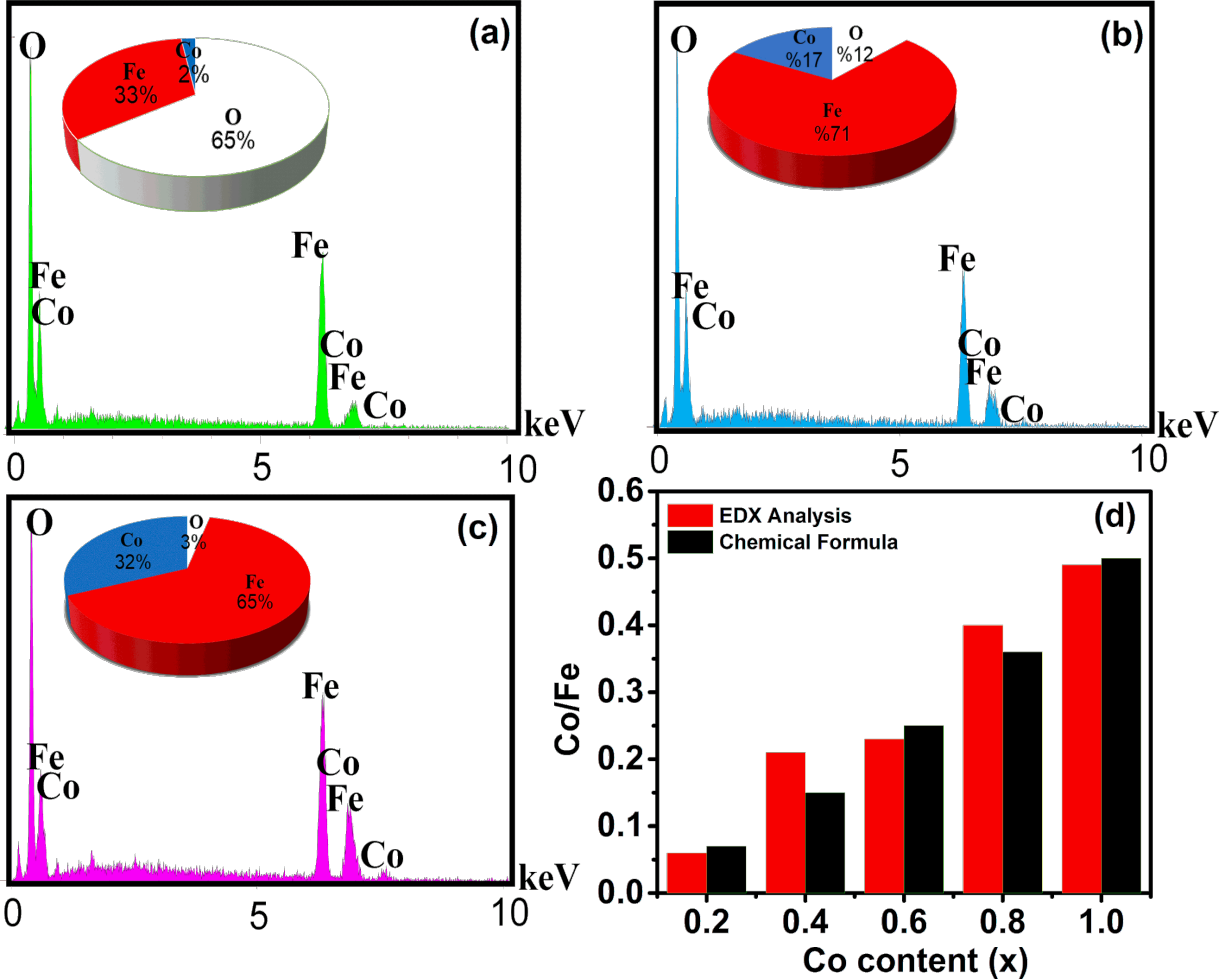
EDX spectra of Co*_x_*Fe_3−_*_x_*O_4_ nanoparticles: (a) *x* = 0.2; (b) *x* = 0.6; and (c) *x* = 1.0. (d) Comparison of the Co/Fe atomic ratio obtained from EDX analysis and the theoretical stoichiometry of all samples.

The qualitative chemical composition of the samples was investigated by using energy-dispersive X-ray spectroscopy (EDX). Figure 4 shows the EDX spectra for the samples with *x* = 0.2, 0.6 and 1. The EDX spectra confirm the presence of Fe, Co and O in the samples. The atomic ratio Co/Fe obtained from EDX is in a good agreement with the theoretical stoichiometry for all samples (Figure 4d).

### Infrared spectra

**Figure 5:**
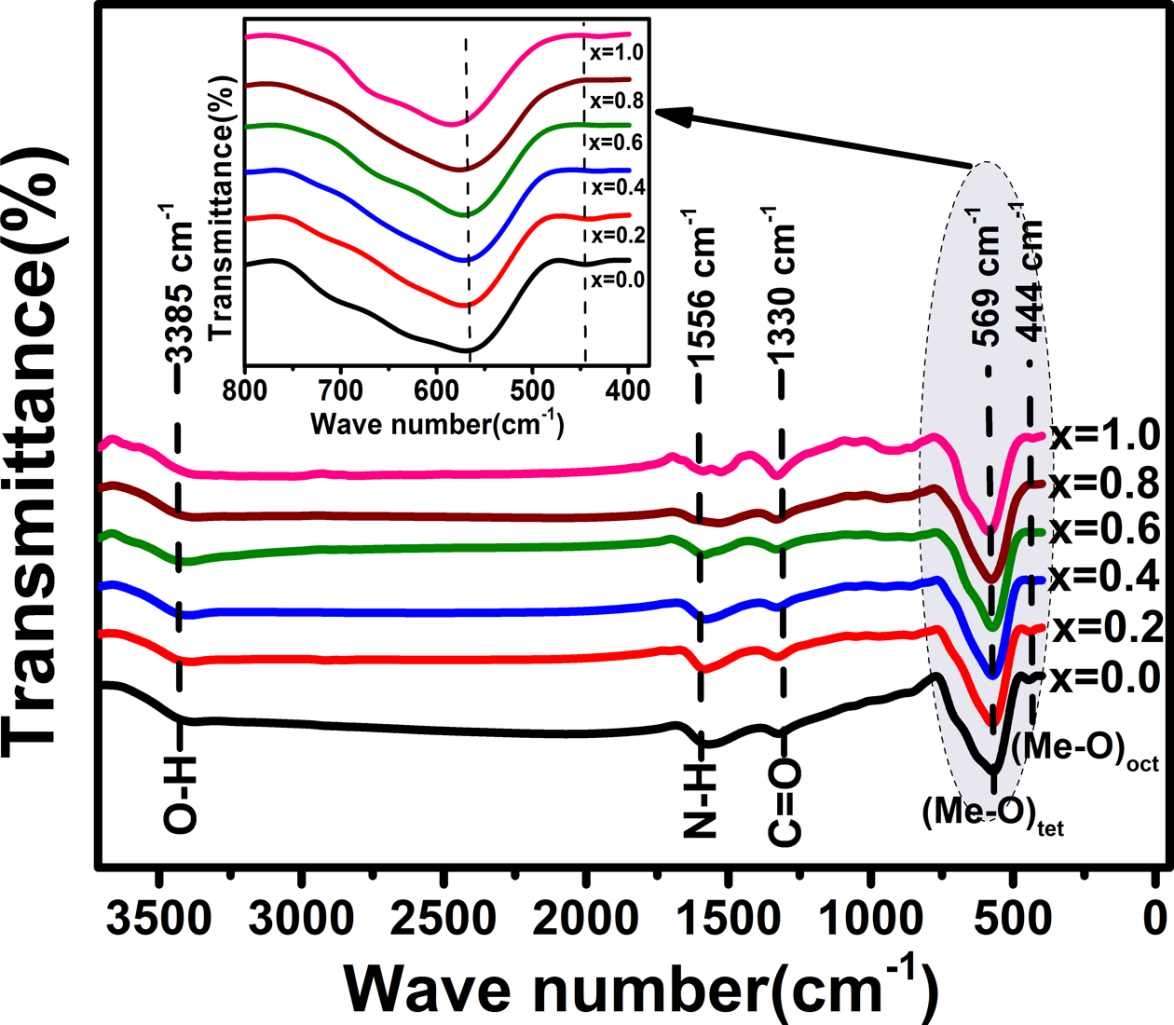
FTIR spectra of Co*_x_*Fe_3−_*_x_*O_4_ nanoparticles with *x* = 0.0, 0.2, 0.4, 0.6, 0.8 and 1.0. The inset is the part of the spectra at frequencies below 1000 cm^−1^.

The formation of the spinel phase and its crystal structure were verified by Fourier-transform infrared (FTIR) spectra measured at 300 K in the wave number range of 400–4000 cm^−1^. Figure 5 shows the FTIR spectra of the samples.

The absorption band observed at around 3385 cm^−1^ is attributed to the vibration mode of the O–H groups in the H_2_O molecules. The peak observed at around 1556 cm^−1^ is ascribed to amide II (NH_2_ deformation, N–H bending) [26] and the absorption peak at around 1330 cm^−1^ is related to the stretching vibration bands of the carboxylate group (C=O) [27]. The latter two peaks (1556 and 1330 cm^−1^) are observed in all samples and can be ascribed to the presence of some impurity in the KBr pellets, which is used for FTIR analysis. Two main absorption bands are observed at frequencies below 1000 cm^−1^. The band around 569 cm^−1^ and the band around 444 cm^−1^ are related to the vibration of metal–oxygen (Me–O) bonds at tetrahedral and octahedral sites, respectively [28–29]. The presence of these two bands confirms the formation of the spinel structure in all the samples.

**Figure 6:**
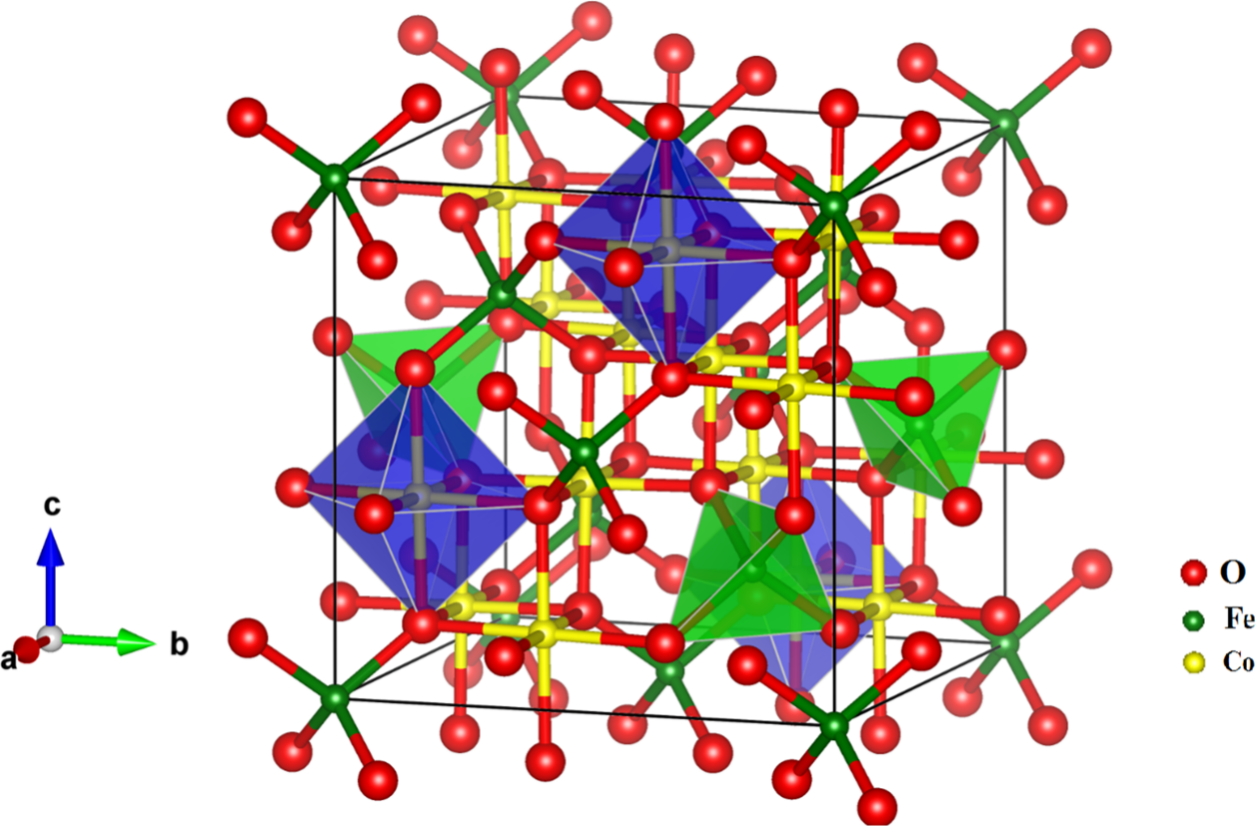
Polyhedral model showing the cubic spinel crystal structure of CoFe_2_O_4_. Green and blue shaded areas correspond to the tetrahedral A-sites and octahedral B-sites, respectively.

The inset in Figure 5 shows that the absorption bands related to the tetrahedral site shift towards higher frequencies with increasing cobalt content. This can be explained by considering that the Co^2+^ ions, being smaller than the Fe^2+^ ions, tend to occupy both the B-sites and the smaller A-sites (see Figure 6). This mixed occupancy in cobalt-substituted magnetite nanoparticles has been confirmed by Mössbauer spectroscopy [30]. Therefore, it is expected that when cobalt ions substitute iron ions at the A-sites, an increasing Me–O bond distance will result. This leads to a weakening of the bond strength and a shift of the peak position towards higher frequencies.

### Thermogravimetric analysis

**Figure 7:**
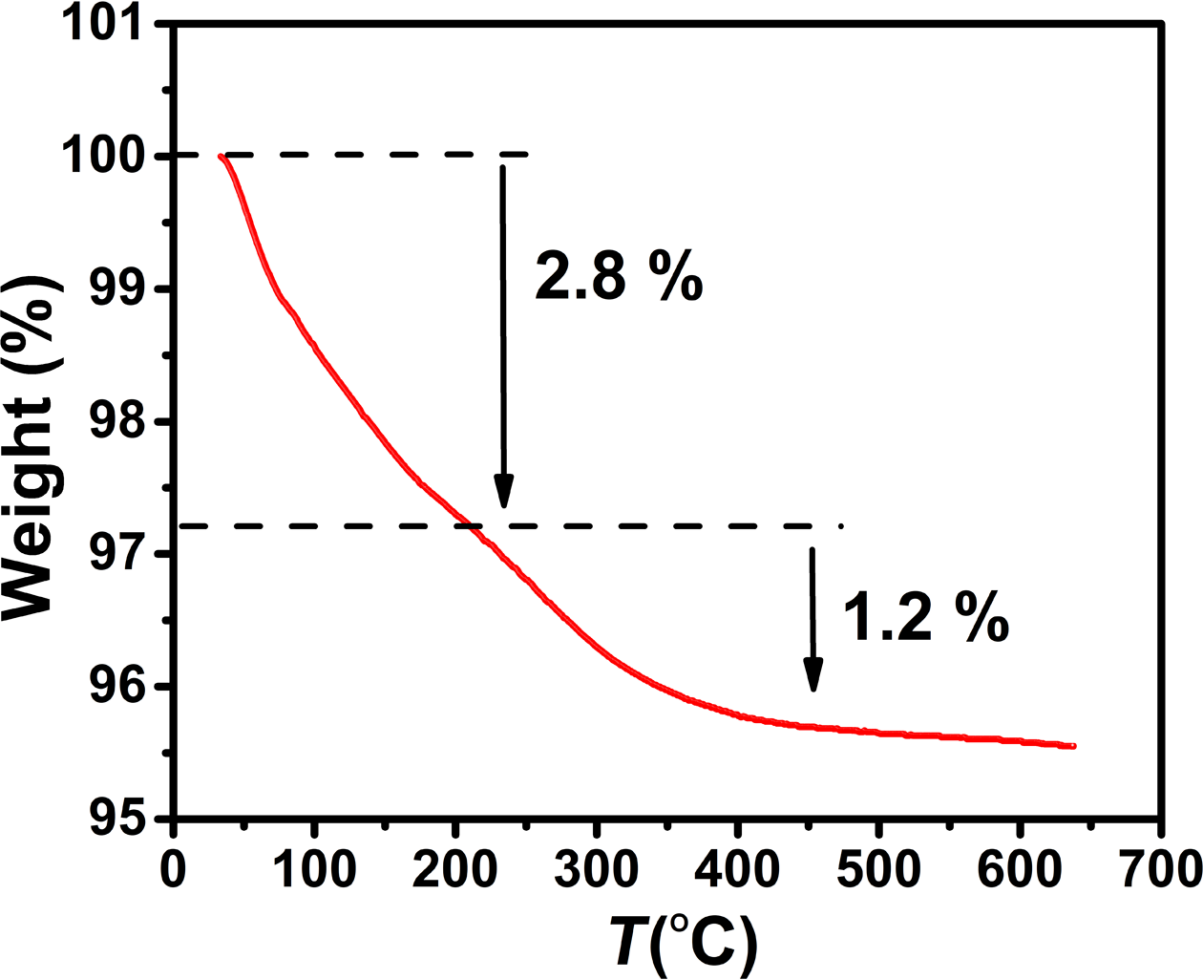
TGA curve of the CoFe_2_O_4_ sample.

The magnetic properties depend on the percentage of the magnetic material (ferrite) in the sample. Hence, the presence of non-magnetic impurities in the CoFe_2_O_4_ samples was checked by thermogravimetric analysis (TGA). Figure 7 shows two weight-loss stages. The first weight loss (about 2.8%), observed in the temperature range of 30–200 °C, is attributed to the vaporization of water from the sample. Since the possible decomposition of spinel ferrite is excluded because of the absence of secondary phases in the XRD patterns, the second weight loss (about 1.2%) between 200 and 500 °C can be attributed to the escape of oxygen atoms from the surface of the NPs [31] and the removal of impurities present in the initial raw materials.

### Magnetic characterization

**Figure 8:**
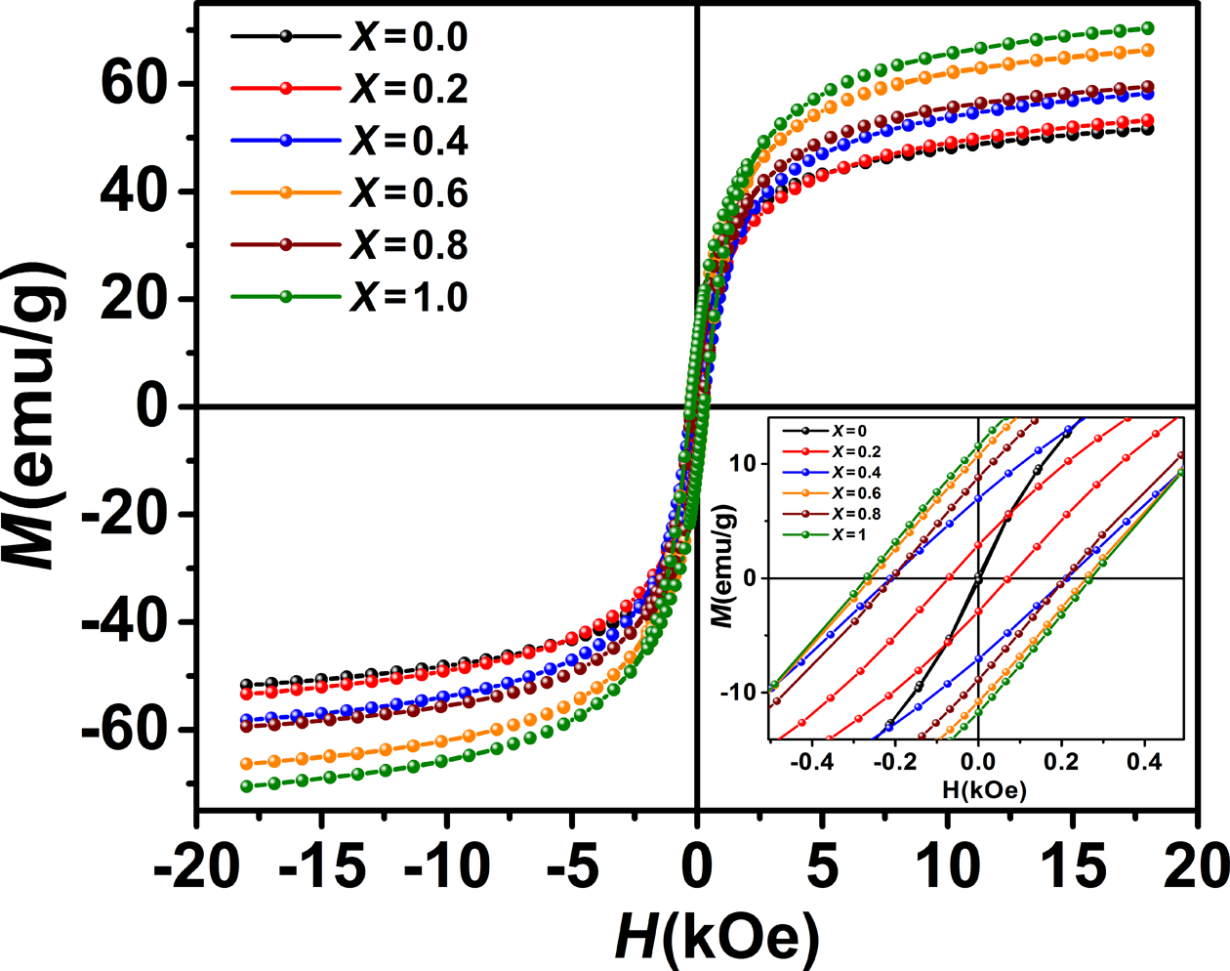
Room-temperature magnetization curves of the Co*_x_*Fe_3−_*_x_*O_4_ samples measured. The inset shows the magnetization behavior at low fields.

Figure 8 shows the room-temperature magnetic hysteresis curves of the samples. The inset of Figure 8 shows that the magnetic behavior of the samples changes from soft ferrite (Fe_3_O_4_) to hard ferrite (CoFe_2_O_4_) as the cobalt content increases.

The values of saturation magnetization (*M*_s_), remanent magnetization (*M*_r_) and coercivity (*H*_c_) obtained from the magnetization curves are given in Table 2.

**Table 2:** Saturation magnetization (*M_S_*), coercivity (*H_C_*) and remanence (*M_r_*) of the Co*_x_*Fe_3−_*_x_*O_4_ samples at room temperature.

parameter	*x* = 0	*x* = 0.2	*x* = 0.4	*x* = 0.6	*x* = 0.8	*x* = 1

*M*_s_ (emu/g)	51.64 ± 0.05	53.30 ± 0.02	58.18 ± 0.01	66.26 ± 0.03	59.42 ± 0.04	70.44 ± 0.11
*H*_c_ (Oe)	3.36 ± 0.65	72.50 ± 0.32	206.97 ± 5.35	258.19 ± 0.99	205.36 ± 2.67	273.24 ± 5.33
*M*_r_ (emu/g)	0.26 ± 0.05	2.91 ± 0.03	7.04 ± 0.05	10.75 ± 0.02	8.84 ± 0.02	11.70 ± 0.07

**Figure 9:**
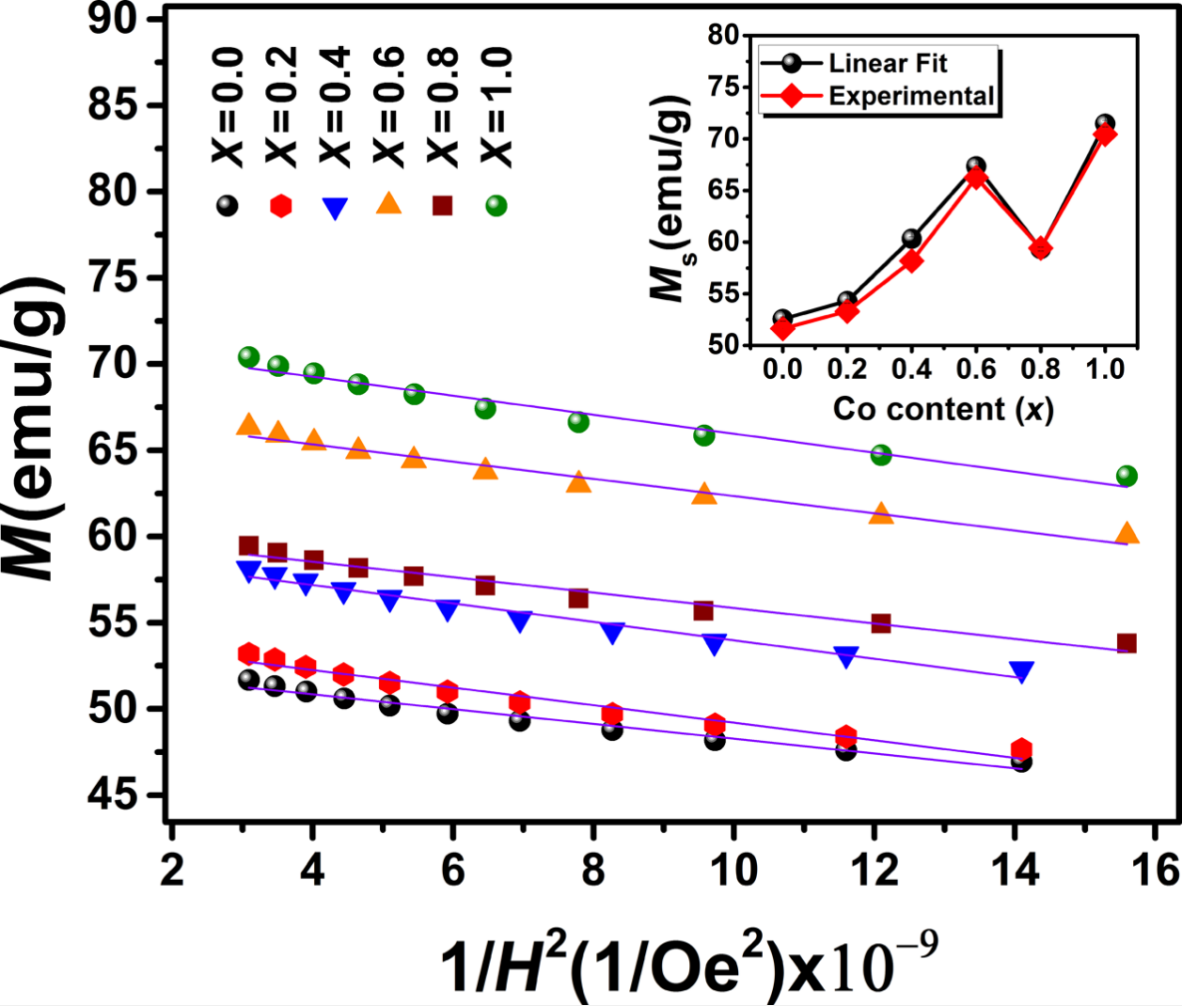
The *M*–1/*H*^2^ dependence of the Co*_x_*Fe_3−_*_x_*O_4_ samples at high field strengths. Experimental data are marked by symbols. The solid lines represent a linear fit of the experimental data using Equation 6. The inset shows the saturation magnetization values obtained from the linear fits and the hysteresis loop curves.

The difference in magnetization of the ferrites NPs is mainly attributed to the difference in particle size [32–33]. The inset of Figure 9 shows that *M*_s_ increases with increasing cobalt content due to increasing the particles size. In fact, the high surface-to-volume ratio in the smaller nanoparticles leads to an increase of the surface effects such as spin disorder and dead layer on the surface, eventually resulting in a decrease of the magnetization. The thickness of the surface dead layer (*t*) equaling to *t* = 0.56 and 0.26 nm for the samples Fe_3_O_4_ and CoFe_2_O_4_*,* respectively, was obtained as follows [34–35]:

[5] $$M_{\text{s}}=M_{\text{b}}\left(1-\frac{6t}{d}\right),$$

where *d* is the particle diameter and *M*_b_ is the bulk saturation magnetization (93 and 80 emu/g for the samples Fe_3_O_4_ and CoFe_2_O_4_, respectively) [3,36].

It is expected that Co increases the magnetic anisotropy in the cubic spinel structure. The effective anisotropy constant (*K*_eff_) of particles was estimated using the law of approach to saturation (LAS), which describes the dependence of the magnetization (*M*) on the applied magnetic field (*H*) at high field strengths (*H* ≫ *H*_c_). According to the LAS, the magnetization near the saturation (*M*_s_) can be expressed as [3,21]:

[6] $$M=M_{\text{s}}\left(1-\frac{b}{H^{2}}\right),$$

where the parameter *b* is associated with the effective anisotropy constant as [21]:

[7] $$K_{\text{eff}}=\mu_{0}M_{\text{s}}\sqrt{\frac{15b}{4}},$$

**Figure 10:**
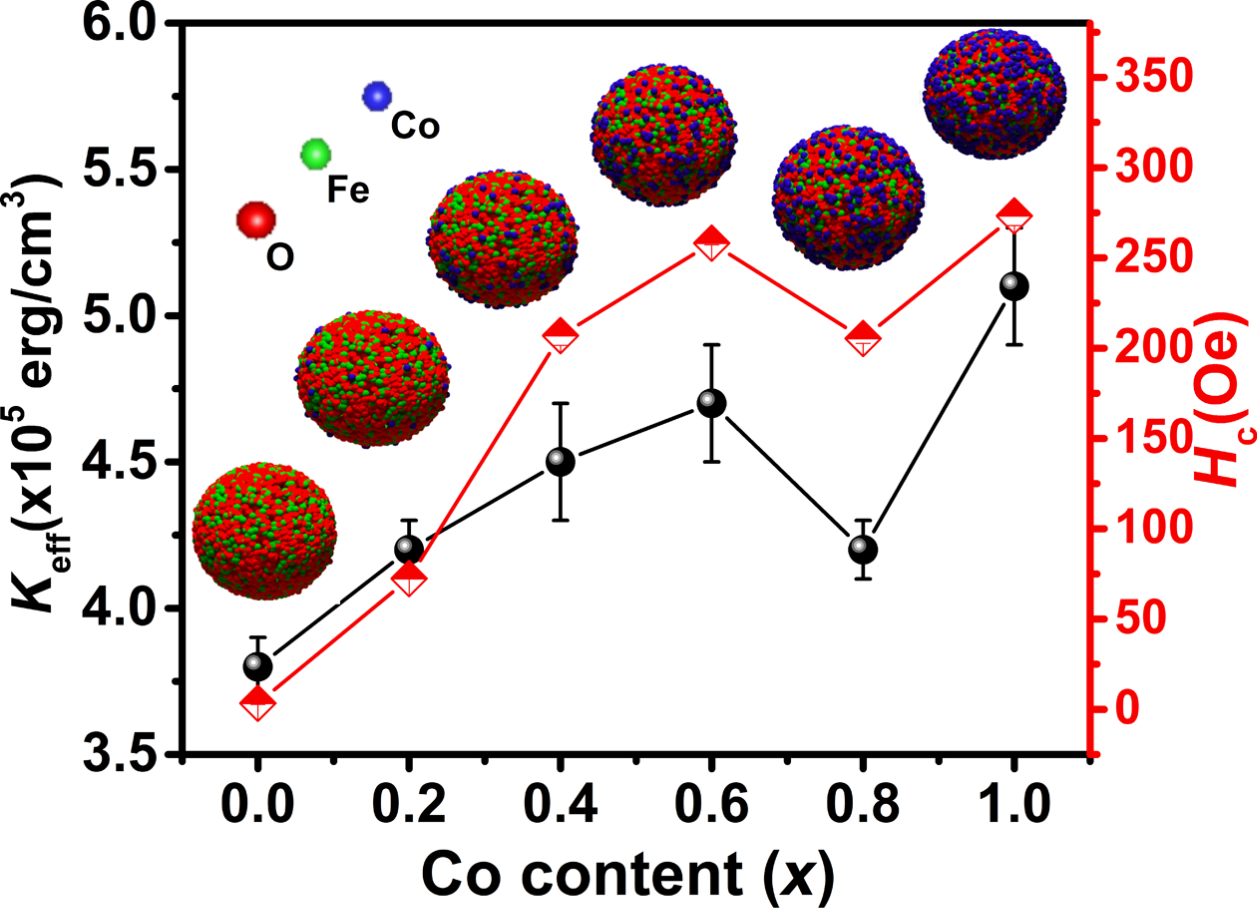
Effective anisotropy constant (*K*_eff_) and coercivity (*H*_c_) of the Co*_x_*Fe_3−_*_x_*O_4_ samples.

To calculate *K*_eff_, the experimental curves of *M* as a function of *1*/*H^2^* were fitted by Equation 6 at high magnetic field strengths (Figure 9). The obtained values of *b* and *M*_s_ were used to calculate *K*_eff_ from Equation 7. The calculated values of *K*_eff_ are presented in Table 3. The result for CoFe_2_O_4_ NPs is in a good agreement with that reported for NPs (ca. 10 nm) of the same compound at room temperature (*K*_eff_ = 3.7 × 10^5^ erg/cm^3^) [17]. Figure 10 shows an increasing magnetic anisotropy with increasing cobalt content. This is due to the gradual occupation of the octahedral sites by cobalt ions and the stronger LS coupling originating from their strong orbital angular momentum [37–38]. The drop of anisotropy in the *x* = 0.8 sample might be due to the decrease of coercivity (because of the smaller size of the single-domain NPs). It is known that the cobalt ions exhibit a strong anisotropy at the octahedral sites of the cubic spinel structure [39]. Also, Mössbauer spectroscopy showed a relatively high number of A-sites occupied by Co^2+^ ions in the *x* = 0.8 sample [40]. Hence, another reason for the sudden drop of the magnetic anisotropy may be attributed to the increasing number of A-sites occupied with Co^2+^ ions, which leads to a reduced anisotropy because of the less anisotropic environment of the A-sites. Deepak et al. [41] observed a sharp decrease in the anisotropy for *x* > 0.6 in Co*_x_*Fe_3−_*_x_*O_4_ nanoparticles. They attributed this effect to Co–Co interactions at high Co concentrations leading to a reduction of anisotropy, while Fe–Co interactions in the lattice increase the magnetocrystalline anisotropy. Figure 10 shows *H*_c_ as a function of the cobalt content. The increase of coercivity is mainly related to the increase of anisotropy. An increase of *H*_c_ with increasing crystallite size has been reported for single-domain NPs [13,42].

**Table 3:** Efective anisotropy constant (*K*_eff_), reverse field (*H*_r_) and mean interaction field (*H*_int_) of the Co*_x_*Fe_3−_*_x_*O_4_ samples.

parameter	*x* = 0.0	*x* = 0.2	*x* = 0.4	*x* = 0.6	*x* = 0.8	*x* = 1.0

*K*_eff_ (× 10^5^ erg(cm^3^)	3.8 ± 0.1	4.2 ± 0.1	4.5 ± 0.2	4.7 ± 0.2	4.2 ± 0.1	5.1 ± 0.2
*H*_r_ (Oe)	197.58	412.03	651.88	726.64	660.12	741.27
*H*_int_ (Oe)	—	−62.36	−77.57	−42.78	−84.27	−87.59

### Remanent magnetization

**Figure 11:**
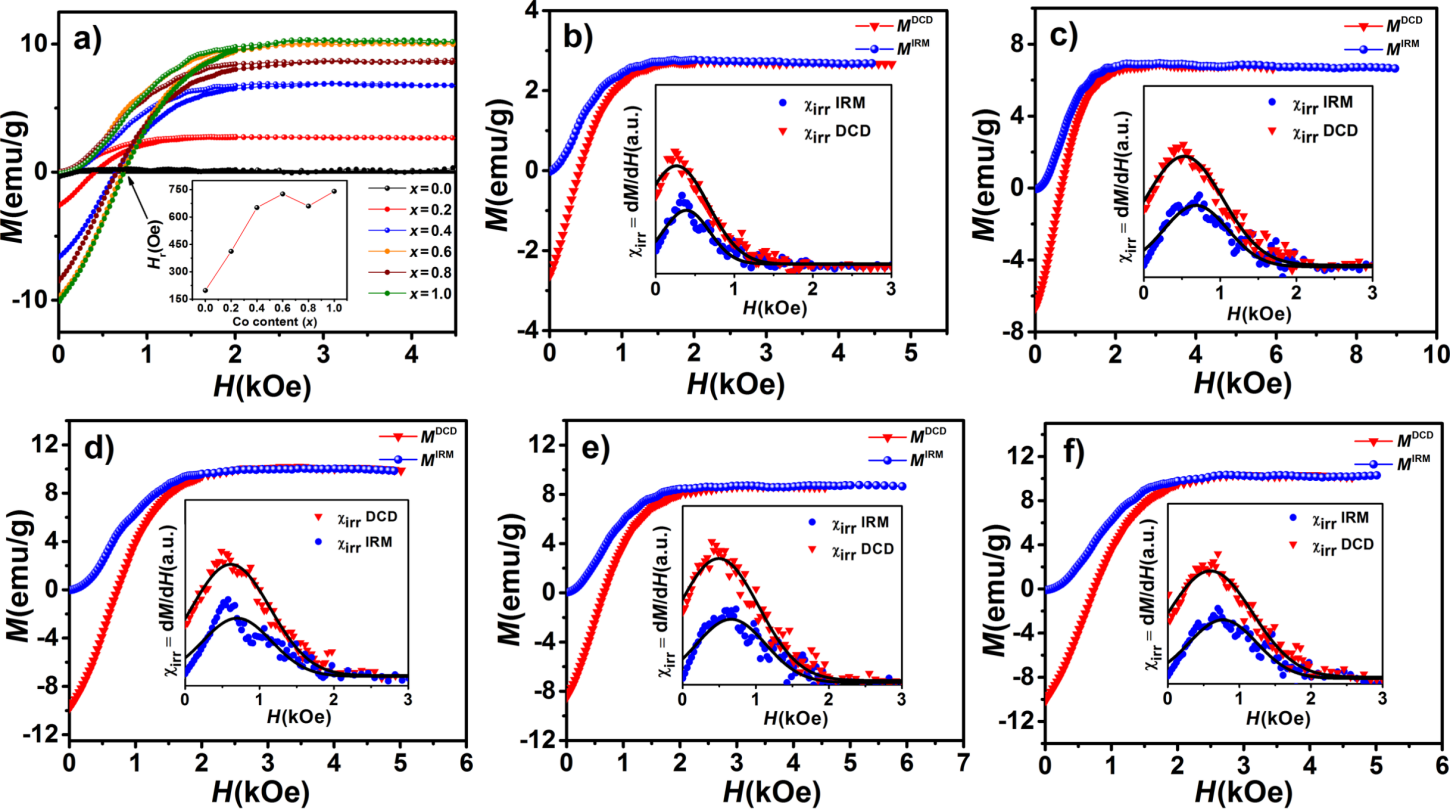
IRM and DCD magnetization curves of (a) all Co*_x_*Fe_3−_*_x_*O_4_ samples and separately for (b) *x* = 0.2, (c) *x* = 0.4, (d) *x* = 0.6, (e) *x* = 0.8 and (f) *x* = 1. The inset in (a) shows the reverse field as a function of *x*. The other insets show the irreversible susceptibility χ_irr_ obtained from *M*^IRM^ and *M*^DCD^ curves.

The analysis of remanent magnetization curves (isothermal remanent magnetization (*M*^IRM^) and direct current demagnetization (*M*^DCD^)) measured at 290 K (Figure 11) allowed us to study the mechanism of interparticle interactions. DCD and IRM curves are given in Figure 11a for all samples.

**Figure 12:**
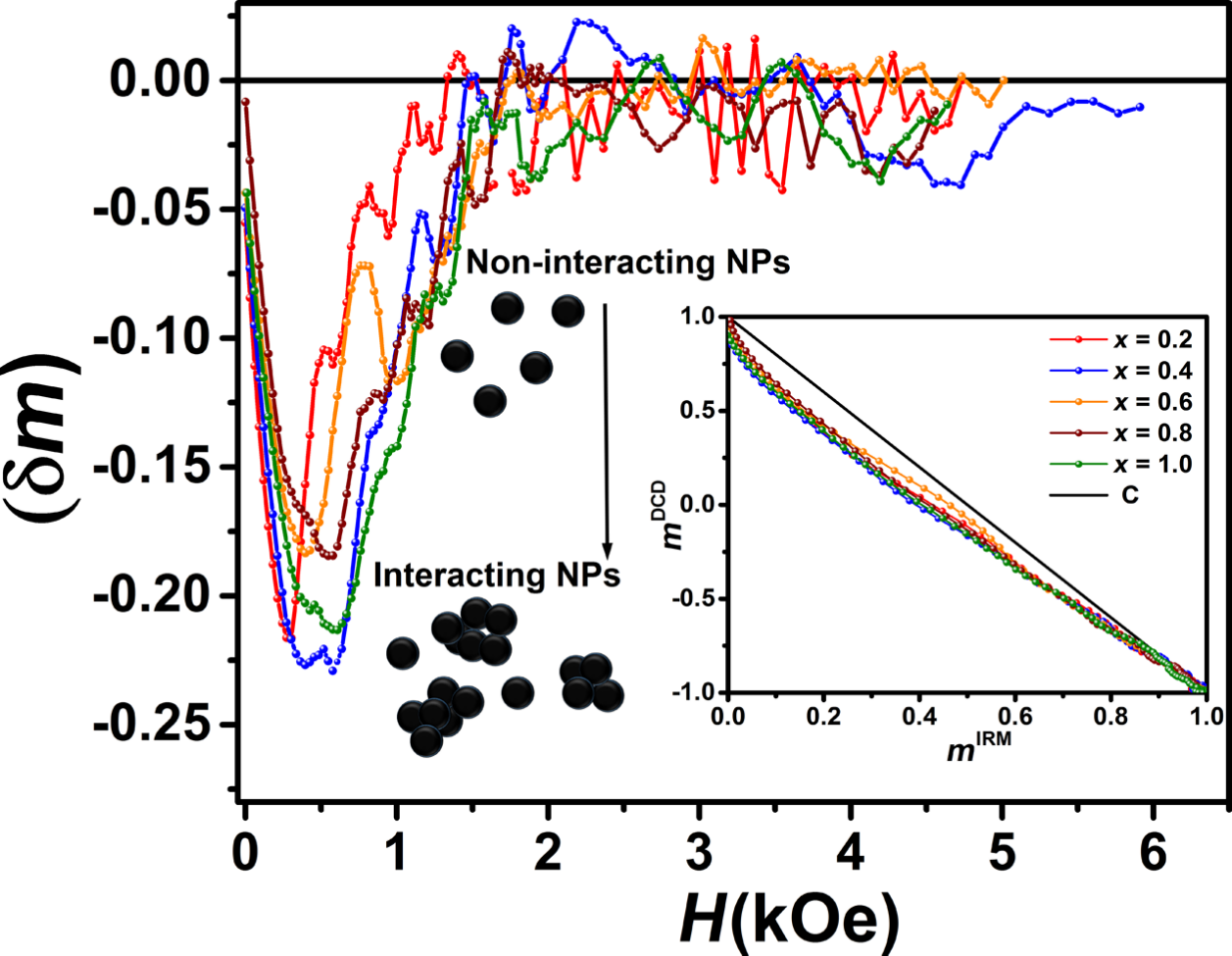
δ*m* as a function of the magnetic field strength measured at room temperature. The inset shows the Henkel plots.

The parameter *H*_r_ shown in the inset of Figure 11a is the remanence coercivity, defined as the reverse field at *M*^DCD^ = 0 [43]. For a system of non-interacting single-domain nanoparticles with uniaxial anisotropy, *M*^IRM^ and *M*^DCD^ curves are related via the Wohlfarth equation [9,13]:

[8] $$m^{\text{DCD}}\left(H\right)=1-2m^{\text{IRM}}\left(H\right),$$

where *m*^DCD^ (*H*) and *m*^IRM^ (*H*) are normalized to the remanence saturation values *M*_s_^IRM^ and *M*_s_^DCD^ of the DCD and the IRM curve, respectively. The interactions in the samples can be quantitatively investigated by the Henkel plot (*m*^DCD^ as a function of *m*^IRM^). According to the Wohlfarth relationship (Equation 8), the Henkel plot of non-interacting nanoparticles should yield a linear function with a slope of −2. Hence, a deviation from linear behavior indicates the presence of interactions between nanoparticles. Kelly et al. showed that in interacting systems, the Henkel plot has a deviation from linearity by an amount of δ*m* = *m*^DCD^(*H*) − (1 − 2*m*^IRM^(*H*)) [44–46]. In particular, a negative peak (a negative deviation of the Henkel plot) in the δ*m* curve indicates the dominance of dipole–dipole interactions, while a positive peak (a positive deviation of the Henkel plot) can be attributed to the dominance of exchange interactions. This is because the dipole–dipole interactions tend to hinder the magnetization (i.e., they have the effect of stabilizing the demagnetized state), while the exchange interactions promote a magnetization. Also, the intensity of the dip of the δ*m* curve depends on the strength of the interactions [9,47–48]. The strength of the interactions can be estimated by calculating the mean interaction field (*H*_int_) defined as [46–47]:

[9] $$H_{\mathrm{int}}=\frac{H^{\text{DCD}}-H^{\text{IRM}}}{2},$$

where *H*^DCD^ and *H*^IRM^ correspond to the peak position of the χ_irr_ (DCD) and the χ_irr_ (IRM) curve, respectively (see Figure 11). In our case, the obtained negative values for *H*_int_ (Table 3) confirmed the presence of dipole–dipole interactions in all samples. The corresponding δ*m* curves and Henkel plots are shown in Figure. 12. The interaction field increases with increasing cobalt content, which can be related to the particle size and the larger magnetic moment of bigger nanoparticles [13,47]. The particle aggregation visible in FE-SEM images shows that the particles are interacting.

The δ*m* plot in Figure 12 indicates that the magnetic interactions between particles are weakest in in the *x* = 0.6 sample. This is in good agreement with the *H*_int_ value and FE-SEM observations. The origin of the low intensity of the δ*m* plot of the *x* = 0.8 sample can be attributed to the smaller particle size, which is clearly visible in the FE-SEM images (Figure 3).

### Magnetic hyperthermia

**Figure 13:**
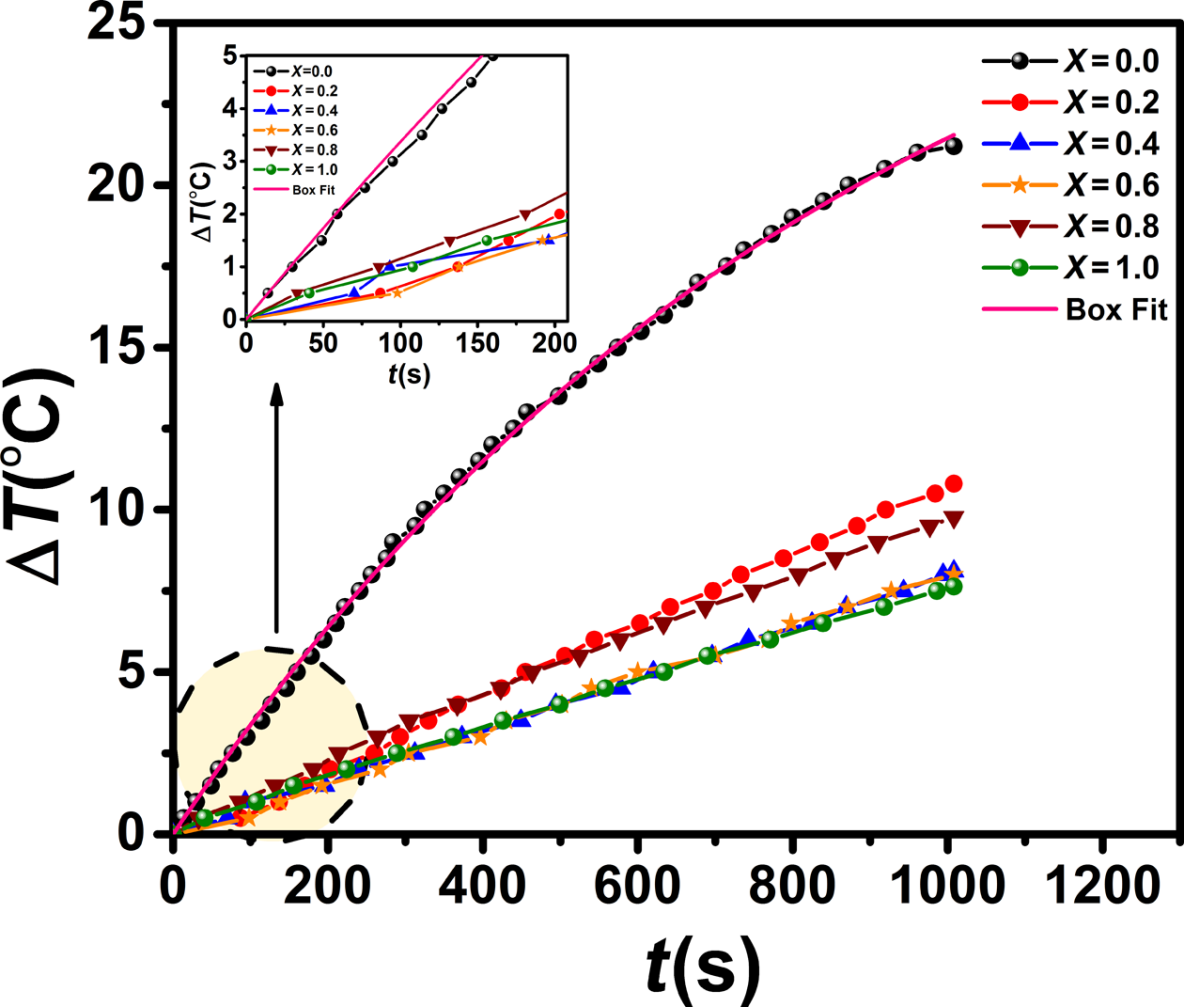
Temperature rise in Co*_x_*Fe_3−_*_x_*O_4_ suspensions in an ac magnetic field (27 Oe, 92 kHz) as a function of the time. The inset shows the temperature rise in the first 200 s. The pink straight line for *x* = 0 is a fit resulting from the Box–Lucas model.

In order to study the heat generation of the nanoparticles for a potential use in magnetic hyperthermia therapy, the samples were dispersed into deionized water at the same concentration (111 mg/mL) and exposed to an ac magnetic field. The increasing temperature as a function of the time was measured. Figure 13 shows a remarkable result, the temperature rise in the *x* = 0 sample with the smallest anisotropy and particle size was much larger than that of the other samples.

Recently, a similar behavior was reported for Ni*_x_*Co_1−_*_x_*Fe_2_O_4_ by Caetano and co-workers [15]. They showed that the heat generation increases with Ni content because of an increase of the minor hysteresis loop area. Our results can be related to the magnetic anisotropy of the NPs. When the coercivity of the NPs is larger than the field amplitude (*H*), the magnetization does not reach complete saturation and exhibits minor loops.

In order to characterize the SAR value of the samples, the curves of the temperature as a function of the time were fitted by the Box–Lucas model, *T*(*t*) = *A*·(1 – *e*^−^*^Bt^*), where *A* is the saturation temperature and *B* is a fit parameter. Here, the product *A*·*B* is the initial rate of the temperature rise. It is equivalent to the ratio d*T*/d*t* in the following equation [49–50]:

[10] $$\text{SAR}=c_{\text{p}}\frac{m_{\text{s}}}{m_{\text{n}}}\frac{\text{d}T}{\text{d}t},$$

where *c*_p_ is the specific heat capacity of the solution (here *c*_p_ = 4.18 J/(g·K) for water), *m*_s_ is the mass of the solution, *m*_s_ is the mass of the nanoparticles and d*T*/d*t* is the initial slope of the heating curves. Figure 13 shows the fit curve using the Box–Lucas model (solid line) for the *x* = 0 sample. The SAR value, or specific loss power (SLP), was then obtained by using Equation 10.

**Table 4:** The values of SAR and ILP of the samples important for the hyperthermia therapy.

parameter	*x* = 0	*x* = 0.2	*x* = 0.4	*x* = 0.6	*x* = 0.8	*x* = 1

SAR (W/g)	1.33	0.44	0.34	0.32	0.49	0.37
ILP (nH·m^2^/kg)	3.13	1.04	0.80	0.75	1.15	0.87

The SAR value is commonly used to characterize the behavior in magnetic hyperthermia. However, it is not an intrinsic property of a given system. It depends on the field amplitude and frequency. Therefore, the intrinsic loss power (ILP) parameter also is useful to compare the heating behavior measured under different values of *f* and *H* [51–52]:

[11] $$\text{ILP}=\frac{\text{SAR}}{H^{2}f}.$$

The values of SAR and ILP decrease with increasing cobalt content. This is because in the samples containing cobalt (*H*_c_ > *H*) the system exhibits minor loops with a slight hysteresis losses.

## Conclusion

In the present paper, we studied the effect of Co doping on the structural, magnetic and hyperthermia properties of Co*_x_*Fe_3−_*_x_*O_4_ nanoparticles. The substitution of Fe by Co leads to an increase of crystallite size, saturation magnetization, coercivity and especially of the magnetic anisotropy of the nanoparticles. Interparticle interactions were disclosed by Henkel plots and δ*m* curves. The negative deviation of the Henkel plots from linearity as well as the negative δ*m* curves indicate a predominance of dipole–dipole interactions in all samples. It was observed that Co doping strongly reduces the specific absorption rate values (to about a fourth) in the samples, despite increasing the magnetic anisotropy, saturation magnetization and particle size. Our results showed that the heat-generation efficiency is highly impacted by the magnetic anisotropy of the nanoparticles.

## Experimental

### Synthesis

Co*_x_*Fe_3−_*_x_*O_4_ nanoparticles were synthesized using a facile co-precipitation method at 80 °C in air. The chemical reaction can be written as follows:

[12]
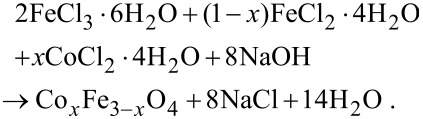


In the first step, stoichiometric amounts of the starting material (see Table 5), including FeCl_3_·6H_2_O (Merck, 99%), FeCl_2_·4H_2_O (Carlo Erba, 99%) and CoCl_2_·4H_2_O (Merck, 99%) were mixed and dissolved in 50 mL of deionized water. Also, NaOH was separately weighed and dissolved in 50 mL deionized water to a concentration of 8.0 M. In the second step, the temperature of the metal-salt solution was raised 80 °C under stirring. Then, the aqueous solution of NaOH was added quickly to the solution at 80 °C. The obtained black slurry was constantly stirred at 80 °C for 30 min. The prepared nanoparticles were washed with deionized water for several times and, finally, dried at room temperature for 24 h.

**Table 5:** Amounts of the starting materials to prepare 1.5 g of Co*_x_*Fe_3−_*_x_*O_4_ nanoparticles.

material	*x* = 0	*x* = 0.2	*x* = 0.4	*x* = 0.6	*x* = 0.8	*x* = 1

*m* (FeCl_3_·6H_2_O) (g)	3.502	3.493	3.484	3.475	3.465	3.456
*m* (FeCl_2_·4H_2_O) (g)	1.288	1.027	0.768	0.511	0.255	0.000
*m* (CoCl_2_·4H_2_O) (g)	0.000	0.307	0.613	0.918	1.220	1.521

### Characterization techniques

Phase purity and crystalline structure of the samples were characterized by using a Philips X′Pert Pro MPDX-ray diffractometer (XRD) with Cu Kα (λ = 0.154 nm) radiation. The diffraction patterns were analyzed using the FullProf-Suite (Version 6.0) software. Thermogravimetric analysis (TGA) was carried out in the temperature range from 30 to 650 °C with a heating rate of 10 °C/min under N_2_ flow using a TGA/SDTA 851 Mettler Toledo thermogravimetric analyzer. Fourier transform infrared (FTIR) spectra of the samples were obtained in the range of 400–4000 cm^−1^ by pressing the powders in KBr pellets. The morphology and elemental chemical composition of the samples were investigated using a Tescan Mira 3 field-emission scanning electron microscope (FE-SEM) equipped with an energy-dispersive X-ray spectrometer. The magnetic properties were studied at room temperature by a custom-built vibrating sample magnetometer (VSM) with a maximum applied field of 18 kOe. The field-dependence of remanent magnetization was measured by following the isothermal remanent magnetization (*M*^IRM^) and direct current demagnetization (*M*^DCD^) protocols. For the *M*^IRM^ measurement, an external field was applied to a demagnetized sample, then it was switched off and the remanent magnetization was measured. This process was repeated, increasing the field up to 18 kOe. In the *M*^DCD^ measurement, the samples were magnetized at −18 kOe. After that, a small field in the opposite direction of magnetization was applied, then the field was switched off and the remanence *M*^DCD^ was measured. This process was repeated increasing the field strength up to +18 kOe. Magnetic hyperthermia properties were studied by using a custom-built setup at a frequency of 92 kHz and a field amplitude of 27 Oe.

## References

[1] Muscas G, Concas G, Laureti S, Testa A M, Mathieu R, De Toro J A, Cannas C, Musinu A, Novak M A, Sangregorio C (2018). Phys Chem Chem Phys.

[2] Pan S, Liu Z, Lu W (2019). Nanotechnology.

[3] Mameli V, Musinu A, Ardu A, Ennas G, Peddis D, Niznansky D, Sangregorio C, Innocenti C, Thanh N T K, Cannas C (2016). Nanoscale.

[4] Usov N A, Nesmeyanov M S, Gubanova E M, Epshtein N B (2019). Beilstein J Nanotechnol.

[5] Kefeni K K, Msagati T A M, Mamba B B (2017). Mater Sci Eng, B.

[6] Deshmukh R, Mehra A, Thaokar R (2017). Beilstein J Nanotechnol.

[7] Gutiérrez L, de la Cueva L, Moros M, Mazarío E, de Bernardo S, de la Fuente J M, Morales M P, Salas G (2019). Nanotechnology.

[8] Serantes D, Simeonidis K, Angelakeris M, Chubykalo-Fesenko O, Marciello M, Del Puerto Morales M, Baldomir D, Martinez-Boubeta C (2014). J Phys Chem C.

[9] De Toro J A, Vasilakaki M, Lee S S, Andersson M S, Normile P S, Yaacoub N, Murray P, Sánchez E H, Muñiz P, Peddis D (2017). Chem Mater.

[10] Routray K L, Saha S, Behera D (2017). Mater Sci Eng, B.

[11] Mohapatra J, Xing M, Liu J P (2018). AIP Adv.

[12] Sathya A, Guardia P, Brescia R, Silvestri N, Pugliese G, Nitti S, Manna L, Pellegrino T (2016). Chem Mater.

[13] Aslibeiki B (2016). Ceram Int.

[14] Lambruschini C, Villa S, Banfi L, Canepa F, Morana F, Relini A, Riani P, Riva R, Silvetti F (2018). Beilstein J Nanotechnol.

[15] Caetano P M A, Albuquerque A S, Fernandez-Outon L E, Macedo W A A, Ardisson J D (2018). J Alloys Compd.

[16] Najafinezhad A, Abdellahi M, Saber-Samandari S, Ghayour H, Khandan A (2018). J Alloys Compd.

[17] Verde E L, Landi G T, Carrião M S, Drummond A L, Gomes J A, Vieira E D, Sousa M H, Bakuzis A F (2012). AIP Adv.

[18] Nemati Z, Alonso J, Martinez L M, Khurshid H, Garaio E, Garcia J A, Phan M H, Srikanth H (2016). J Phys Chem C.

[19] Le A-T, Giang C D, Tam L T, Tuan T Q, Phan V N, Alonso J, Devkota J, Garaio E, García J Á, Martín-Rodríguez R (2016). Nanotechnology.

[20] Orozco-Henao J M, Coral D F, Muraca D, Moscoso-Londoño O, Mendoza Zélis P, Fernandez van Raap M B, Sharma S K, Pirota K R, Knobel M (2016). J Phys Chem C.

[21] Nemati Z, Salili S M, Alonso J, Ataie A, Das R, Phan M H, Srikanth H (2017). J Alloys Compd.

[22] Barrera G, Coisson M, Celegato F, Raghuvanshi S, Mazaleyrat F, Kane S N, Tiberto P (2018). J Magn Magn Mater.

[23] Fantechi E, Innocenti C, Albino M, Lottini E, Sangregorio C (2015). J Magn Magn Mater.

[24] Anjum S, Tufail R, Rashid K, Zia R, Riaz S (2017). J Magn Magn Mater.

[25] Luo Y-R (2007). Comprehensive Handbook of Chemical Bond Energies.

[26] Kalska-Szostko B, Wykowska U, Satula D, Nordblad P (2015). Beilstein J Nanotechnol.

[27] Yang M-H, Yuan S-S, Chung T-W, Jong S-B, Lu C-Y, Tsai W-C, Chen W-C, Lin P-C, Chiang P-W, Tyan Y-C (2014). BioMed Res Int.

[28] Sharma R, Thakur P, Sharma P, Sharma V (2017). J Alloys Compd.

[29] Aslibeiki B (2014). Curr Appl Phys.

[30] Kombaiah K, Vijaya J J, Kennedy L J, Bououdina M, Al Najar B (2018). J Alloys Compd.

[31] Wang Z, Wang W, Zhang L, Jiang D (2016). Catal Sci Technol.

[32] Aslibeiki B, Kameli P, Ehsani M H, Salamati H, Muscas G, Agostinelli E, Foglietti V, Casciardi S, Peddis D (2016). J Magn Magn Mater.

[33] Kumar K, Loganathan A (2017). Mater Sci Eng, B.

[34] Aslibeiki B, Varvaro G, Peddis D, Kameli P (2017). J Magn Magn Mater.

[35] Virumbrales-del Olmo M, Delgado-Cabello A, Andrada-Chacón A, Sánchez-Benítez J, Urones-Garrote E, Blanco-Gutiérrez V, Torralvo M J, Sáez-Puche R (2017). Phys Chem Chem Phys.

[36] Aslibeiki B, Ehsani M H, Nasirzadeh F, Mohammadi M A (2017). Mater Res Express.

[37] Li D, Yun H, Diroll B T, Doan-Nguyen V V T, Kikkawa J M, Murray C B (2016). Chem Mater.

[38] Sharma R, Thakur P, Kumar M, Thakur N, Negi N S, Sharma P, Sharma V (2016). J Alloys Compd.

[39] Betancourt-Galindo R, Ayala-Valenzuela O, García-Cerda L A, Rodríguez Fernández O, Matutes-Aquino J, Ramos G, Yee-Madeira H (2005). J Magn Magn Mater.

[40] Li X, Kutal C (2003). J Alloys Compd.

[41] Deepak F L, Bañobre-López M, Carbó-Argibay E, Cerqueira M F, Piñeiro-Redondo Y, Rivas J, Thompson C M, Kamali S, Rodríguez-Abreu C, Kovnir K (2015). J Phys Chem C.

[42] Ghunaim R, Scholz M, Damm C, Rellinghaus B, Klingeler R, Büchner B, Mertig M, Hampel S (2018). Beilstein J Nanotechnol.

[43] Cannas C, Musinu A, Ardu A, Orrù F, Peddis D, Casu M, Sanna R, Angius F, Diaz G, Piccaluga G (2010). Chem Mater.

[44] Kelly P E, O'Grady K, Mayo P I, Chantrell R W (1989). IEEE Trans Magn.

[45] Fabris F, Xing Y T, Franceschini D F, Sanchez D R, Alzamora M, Nunes W C (2017). J Appl Phys.

[46] Coral D F, Mendoza Zélis P, Marciello M, Morales M d P, Craievich A, Sánchez F H, Fernández van Raap M B (2016). Langmuir.

[47] Lavorato G C, Peddis D, Lima E, Troiani H E, Agostinelli E, Fiorani D, Zysler R D, Winkler E L (2015). J Phys Chem C.

[48] Ojha S, Nunes W C, Aimon N M, Ross C A (2016). ACS Nano.

[49] Jadhav S V, Kim B M, Lee H Y, Im I C, Rokade A A, Park S S, Patil M P, Kim G D, Yu Y S, Lee S H (2018). J Alloys Compd.

[50] Ralandinliu Kahmei R D, Borah J P (2019). Nanotechnology.

[51] Cruz M M, Ferreira L P, Ramos J, Mendo S G, Alves A F, Godinho M, Carvalho M D (2017). J Alloys Compd.

[52] Zargar T, Kermanpur A (2017). Ceram Int.

